# Understanding the effect of alternative splicing in the folding and function of the second PDZ from Protein Tyrosine Phosphatase-BL

**DOI:** 10.1038/srep09299

**Published:** 2015-03-19

**Authors:** Eva Di Silvio, Angelo Toto, Daniela Bonetti, Angela Morrone, Stefano Gianni

**Affiliations:** 1 Istituto Pasteur - Fondazione Cenci Bolognetti and Istituto di Biologia e Patologia Molecolari del CNR, Dipartimento di Scienze Biochimiche “A. Rossi Fanelli”, Sapienza Università di Roma, P.le A. Moro 5, 00185, Rome, Italy; 2 Department of Chemistry, University of Cambridge, Lensfield Road, Cambridge CB2 1EW, United Kingdom

## Abstract

PDZ domains are the most prominent biological structural domains involved in protein-protein interactions in the human cell. The second PDZ domain of the protein tyrosine phosphatase BL (PDZ2) interacts and binds the C-termini of the tumour suppressor protein APC and of the LIM domain-containing protein RIL. One isoform of PDZ2 (PDZ2as) involves an alternative spliced form that exhibits an insertion of 5 residues in a loop. PDZ2as abrogates binding to its partners, even if the insertion is directly located in its binding pocket. Here, we investigate the folding and function of PDZ2as, in comparison to the previously characterized PDZ2 domain. Data reveal that, whilst the thermodynamic stability of PDZ2as appears as nearly identical to that of PDZ2, the insertion of 5 amino acids induces formation of some weak transient non-native interactions in the folding transition state, as mirrored by a concomitant increase of both the folding and unfolding rate constants. From a functional perspective, we show that the decrease in affinity is caused by a pronounced decrease of the association rate constants (by nearly ten fold), with no effect on the microscopic dissociation rate constants. The results are briefly discussed in the context of previous work on PDZ domains.

The PDZ domain family represents the most abundant protein interaction modules in human, characterized by more than 200 different PDZ domains^1^,^2^,^3^. The PDZ domains exert their function by binding to a specific target via the recognition of a short stretch of amino acids (the so-called PDZ motif), located typically, but not exclusively^4^, at the carboxyl terminus of polypeptide^5^. From a structural point of view, the three-dimensional architecture of the PDZ domains corresponds to a globular fold of about 90 amino acid residues, composed of six β-strands and two α-helices; the six β-strands form two antiparallel β-sheets stacked onto each other (Figure 1)^6^. The binding pocket is universally located between helix α2 and strand β2^3^. In addition to their role in scaffolding, several studies suggested that PDZ domains might also display a regulatory function^2^,^7^,^8^,^9^. Furthermore, these protein family has been reported to display allosteric properties, which have been ascribed both to conformational^10^ and to dynamic changes^11^,^12^, and have been mapped structurally both by molecular dynamics, NMR and site-directed mutagenesis^10^,^11^,^13^,^14^,^15^,^16^.

The protein tyrosine phosphatase BL (PTP-BL) is a cytosolic protein containing a tandem of five PDZ domains (labelled PDZ1-PDZ5). The PDZ domains of PTP-BL have been shown to interact with several proteins^17^,^18^,^19^,^20^,^21^,^22^,^23^. The functional links within this web of interactions have yet to be described in a coherent scheme. Among the five PDZ domain of PTP-BL, the second PDZ domain (PDZ2) has been reported to interact with the proteins RIL and APC and the interactions with the PDZ motifs from these partners have been previously characterized from a structural and thermodynamic perspective^10^,^21^,^23^,^24^,^25^,^26^.

Interestingly, PDZ2 can be expressed into two alternative spliced forms - a short PDZ2 domain of 93 residues or an alternatively spliced isoform containing 98 residues^20^. The alternatively spliced form (PDZ2as) exhibits an insertion of 5 residues (VLFDK) at the beginning of the β2–β3 loop that abrogates binding to the C-terminal peptide of APC and RIL^27^,^28^. Despite it has been previously observed that the β2–β3 loop may have a role in fine tuning the affinity of PDZ domains to their target^29^,^30^, the remarkable change in affinity induced by the alternative splicing is somewhat surprising, the binding pocket being physically located at the interface between helix α2 and strand β2. This finding appears to confirm the existence of long range effects in PDZ domains. Detailed NMR analysis of PDZ2as, in comparison with PDZ2, suggested binding capabilities to be abrogated by: i) a detectable conformational change in PDZ2as, displaying a closed binding pocket with respect of PDZ2; ii) the replacement of the GG hinge region at the beginning of the β2–β3 loop (a conserved feature in the PDZ fold) by the more conformationally restricted VL sequence; iii) a global destabilisation of the domain^27^,^28^. The conformational changes induced by the insertion of the 5 residues in PDZ2as appear very similar to those observed between the free state of PDZ2 and its bound state to APC (Figure 1)^10^,^28^.

Here, we report a complete characterization of the folding and binding properties of PDZ2as, using stopped-flow and temperature-jump experiments carried out under different experimental conditions. In particular, we show that on the contrary of what previously suggested^28^, the thermodynamic stability of PDZ2as, defined as the difference in free energy between the native and the denatured states, appears as nearly identical to that of PDZ2. A close investigation of the folding kinetics, however, reveals a more complex scenario and suggests that the insertion of 5 amino acids in the β2–β3 loop induces formation of some non-native interactions in the transition state, as mirrored by a concomitant increase of both the folding and unfolding rate constants. From a functional perspective, we describe the binding properties of PDZ2as to APC and RIL peptides and show that the decrease in affinity is caused by a remarkable decrease of the association rate constants (by nearly ten fold), with no effect on the microscopic dissociation rate constants. Finally, we briefly discuss our experiments in the context of previous work on PDZ2as.

## Results

**Figure 1:**
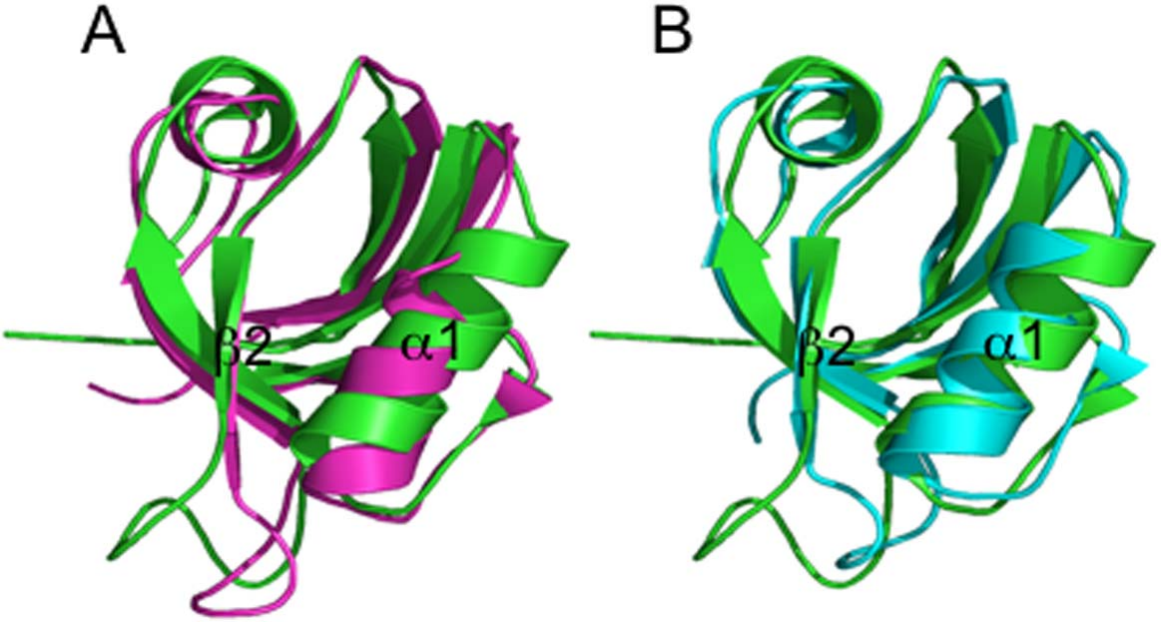
Three dimensional structure of PDZ2as in comparison with the free and bound form of PDZ2. The structure of PDZ2as (pdb code 1OZI) is indicated in green and it is structurally aligned with the free PDZ2 (pdb code 1GM1; purple) and complexed PDZ2 (from the pdb code 1VJ6). It is evident that the binding of the peptide induces a rotation of the α1 helix with respect to β2, thus closing the binding site. Such a rotation is also observed in the free structure of PDZ2as, that resembles the structure of the bound state of PDZ2.

**Figure 2:**
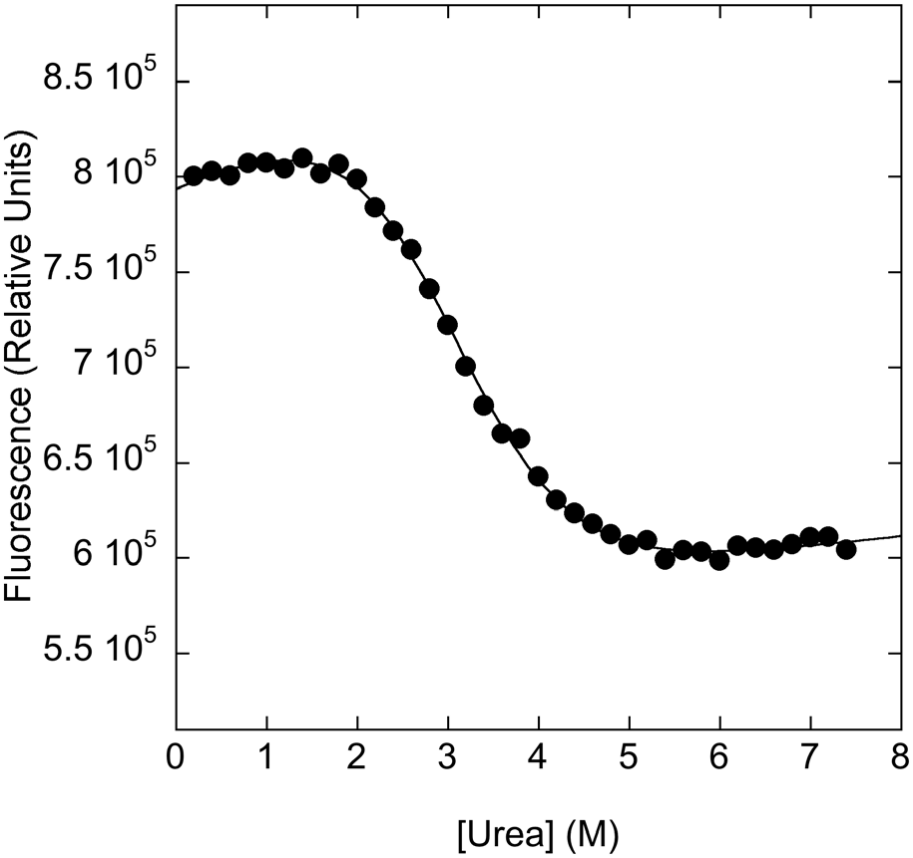
Equilibrium unfolding of pWT43 PDZ2as. Urea induced denaturation measured at pH 7.2 in 50 mM sodium phosphate buffer and 25°C in the presence of 0.4 M sodium sulphate, followed by fluorescence (3 μM protein concentration). The excitation wavelength was 280 nm. Fluorescence emission was recorded between 300 and 400 nm. Data in the Figure refers to 350 nm. Line is the best fit to a two-state model. The unfolding free energy in water derived from two-state analysis is 3.2 ± 0.3 kcal mol^−1^ with an observed *m*-value of 1.1 ± 0.1 kcal mol^−1^ M^−1^. Analysis of the data at different wavelengths returned similar thermodynamics parameters.

In order to study the folding mechanism of PDZ2as, in analogy to our previous work on PDZ domains, we produced a fluorescent pseudo-wild-type, namely Y43W (pWT43) (Figure 1). Furthermore, we compared the thermodynamic stability of the two proteins at similar experimental conditions. Urea-induced equilibrium denaturation of pWT43 PDZ2as measured at 25°C, pH 7.2 in 50 mM sodium phosphate buffer and in the presence of 0.4 M sodium sulphate is reported in Figure 2. The observed transition, followed by decrease in Trp emission, conforms to simple two-state behavior, suggesting the absence of stable equilibrium intermediate(s)^31^. The unfolding free energy in water derived from two-state analysis is 3.2 ± 0.3 kcal mol^−1^. The observed *m*-value, reflecting the difference in accessible surface area between the native and denatured state^32^, is 1.1 ± 0.1 kcal mol^−1^ M^−1^, consistent with a protein of about 98 amino acids^33^ and, therefore, suggesting lack of residual structure in the denatured state of PDZ2as. On the contrary of what previously suggested by NMR experiments^28^, it appears that the apparent stability of pWT43 PDZ2as, defined as the change in free energy between the native and the denatured state, is nearly identical to that observed for pWT43 PDZ2, corresponding to a value of 3.6 ± 0.2 kcal mol^−1^ 34.

**Figure 3:**
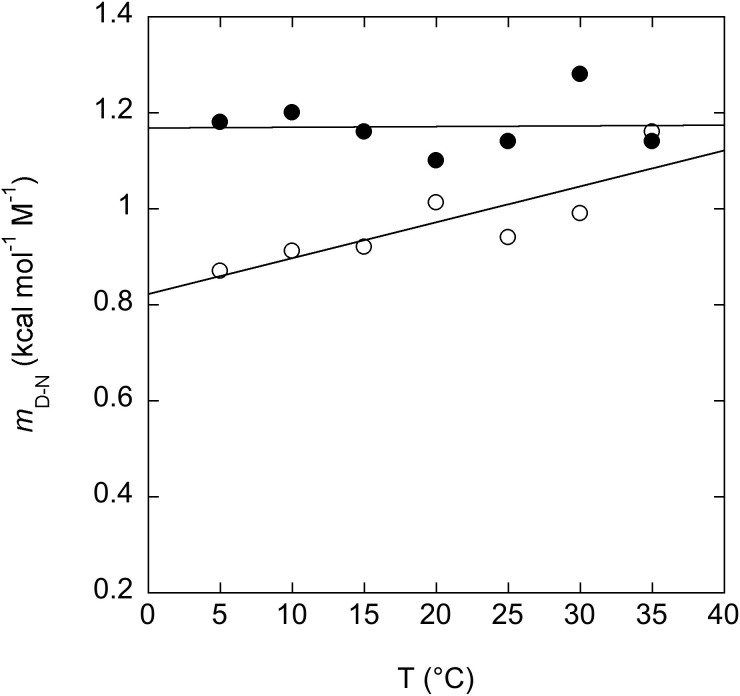
Dependence of the unfolding *m*-value from temperature in the folding of pWT43 PDZ2as. Data refer to different equilibrium denaturations recorded for pWT43 PDZ2as (filled circles) and pWT43 PDZ2 (empty circles) at different temperatures ranging from 5 to 35°C at pH 7.2 in 50 mM sodium phosphate buffer and in the presence of 0.4 M sodium sulphate. The line is the best fit to a linear equation. As discussed in the text, whilst in the case of pWT43 PDZ2 we could detect a dependence of the observed *m*-value from temperature, in agreement with what reported in reference^26^, the *m*-values of pWT43 PDZ2as were insensitive to temperature. This observation indicates that the insertion of the 5 amino acid in the β2–β3 loop has an effect on the residual structure of the denatured state of this PDZ domain, preventing the formation of the residual structures at lower temperatures.

Previous equilibrium studies on PDZ2 suggested that this domain displays a residual structure in its denatured state that can be tuned by temperature^26^, as mirrored by a pronounced temperature dependence of its associated denaturation *m*-values. In an effort to compare the denatured state properties of PDZ2 and PDZ2as, we therefore performed a complete set of equilibrium denaturations at different temperatures. Interestingly, it appears that whilst PDZ2 displays clear temperature dependence, with observed *m*-values decreasing with decreasing temperatures (typically associated with a compaction of the denatured state with decreasing temperatures), in the case of PDZ2as the *m*-values were found to be insensitive on temperature (Figure 3). This observation suggests that the insertion of the 5 amino acid in the β2–β3 loop has an effect on the residual structure of the denatured state of this PDZ domain, preventing the formation of the residual structures at lower temperatures. The observation of a change in the residual structure of the denatured state in PDZ2as implies that a direct comparison between the thermodynamic stability of PDZ2 and PDZ2as from urea denaturation experiments should be taken cautiously, as it is likely that both the native and denatured states stability are affected by the insertion of 5 amino acids.

**Figure 4:**
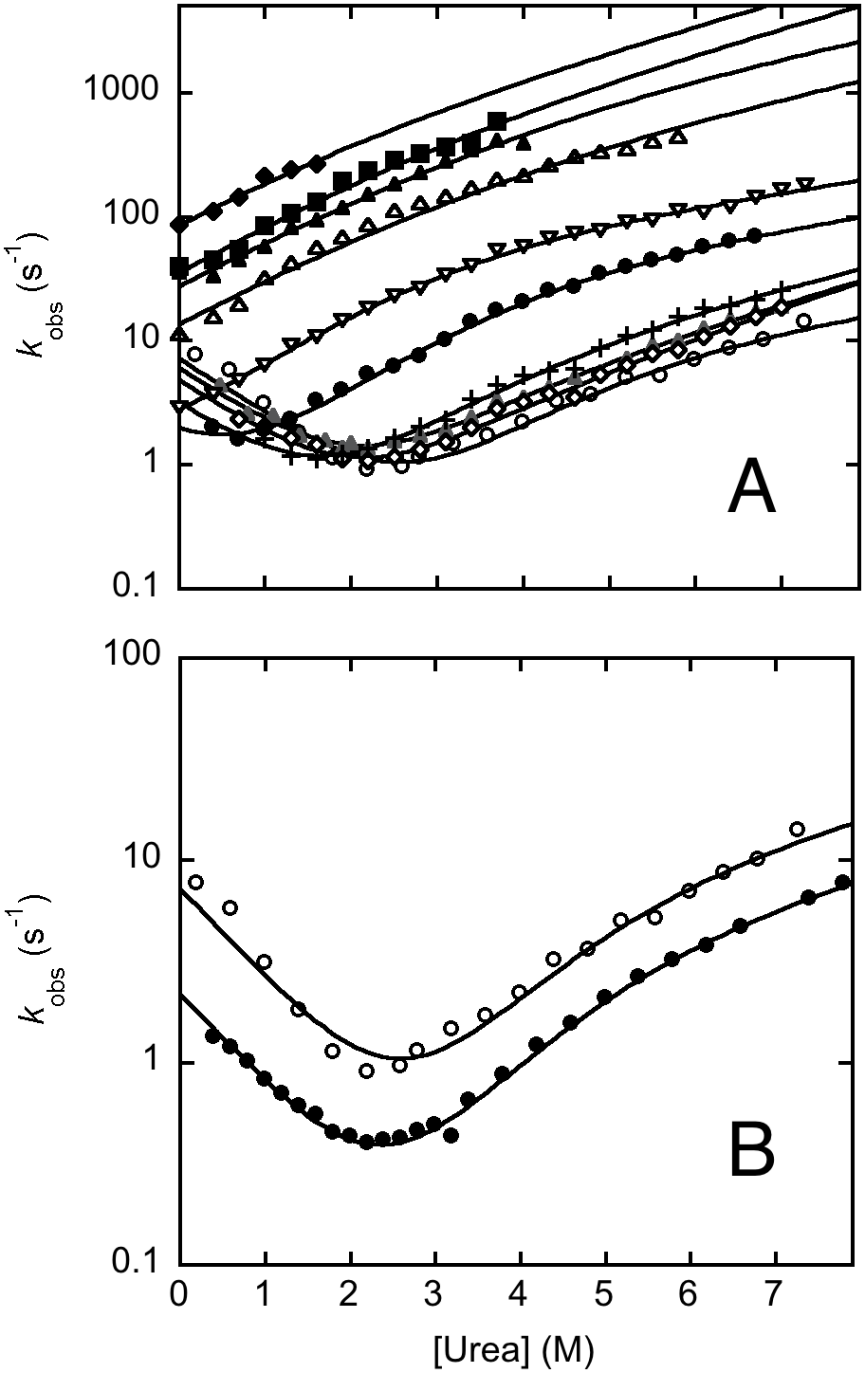
Folding kinetics of pWT43 PDZ2as. Panel A. Semilogarithmic plot of the observed folding and unfolding rate constants as a function of [urea] measured at different pH values and 25°C (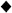, pH 2.0; 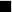, pH 2.8; 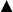, pH 3.0; 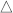, pH 3.4; 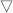, pH 3.8; 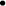, pH 4.7; +, pH 5.5; 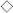, pH 6.3; 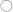, pH 7.0; 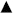, pH 8.0). The lines are the best fit to a three state model as formalized in Equation 2; calculated parameters are listed in Table 1. Panel B. Comparison of the chevron plot of pWT43 PDZ2as (empty circles) and pWT43 PDZ2 (filled circles) measured at pH 7.2 in 50 mM sodium phosphate buffer and 25°C. pWT43 PDZ2as appears to fold and unfold about three times faster than pWT43 PDZ2, while displaying a nearly identical stability. This suggests that the insertion of 5 amino acids in pWT43 PDZ2as induces some non-native interactions in both the early and the late transition states TS1 and TS2.

Addressing the effect of alternative splicing in the mechanism of folding of PDZ2 demands kinetic experiments. Thus, in analogy to our earlier experiments^25^,^35^,^36^,^37^, we performed a complete analysis of the folding and unfolding kinetics at different experimental conditions. The folding and unfolding kinetics of pWT43 PDZ2as were investigated at several pH values ranging from 2.1 to 8.0 and at 25°C. Folding and unfolding reactions were respectively initiated by rapidly mixing, in the stopped-flow apparatus, the native protein with solutions of urea at different concentrations (unfolding) or by rapidly diluting the unfolded protein in the presence of urea, with refolding buffer at the desired final urea concentration (refolding). Due to the low stability of the protein, below pH 3.8, the initial native protein was kept at physiological pH and unfolding experiments where triggered by a simultaneous urea and pH jump. In all cases, folding and unfolding time courses were both fitted satisfactorily to a single exponential decay at any final denaturant concentration. A semi-logarithmic plot of the observed folding/unfolding rate constant versus denaturant concentration (chevron plot) measured at various pH values is reported in Figure 4A. A comparison between the chevron plots of pWT43 PDZ2 and pWT43 PDZ2as at pH 7.2 is reported in Figure 4B. Overall, pWT43 PDZ2as appears to fold and unfold about three times faster than pWT43 PDZ2, while displaying a nearly identical stability. Interestingly, whereas the logarithm of the observed refolding rate constant decreases linearly with increasing denaturant concentration (as seen at pH > 5.5), the observed unfolding rate constants present a downward curvature as a function of urea (roll-over effect). This effect has been already addressed in various PDZ domains^5^,^25^,^35^,^36^,^37^, including PDZ2, and has been associated with the presence of two consecutive transition states, namely a denatured like transition state TS1 and a more native like transition state TS2. At this stage, data did not reveal the presence of a third transition state TS3, which has been recently detected for the second PDZ domain from SAP95^38^.

**Figure 5:**
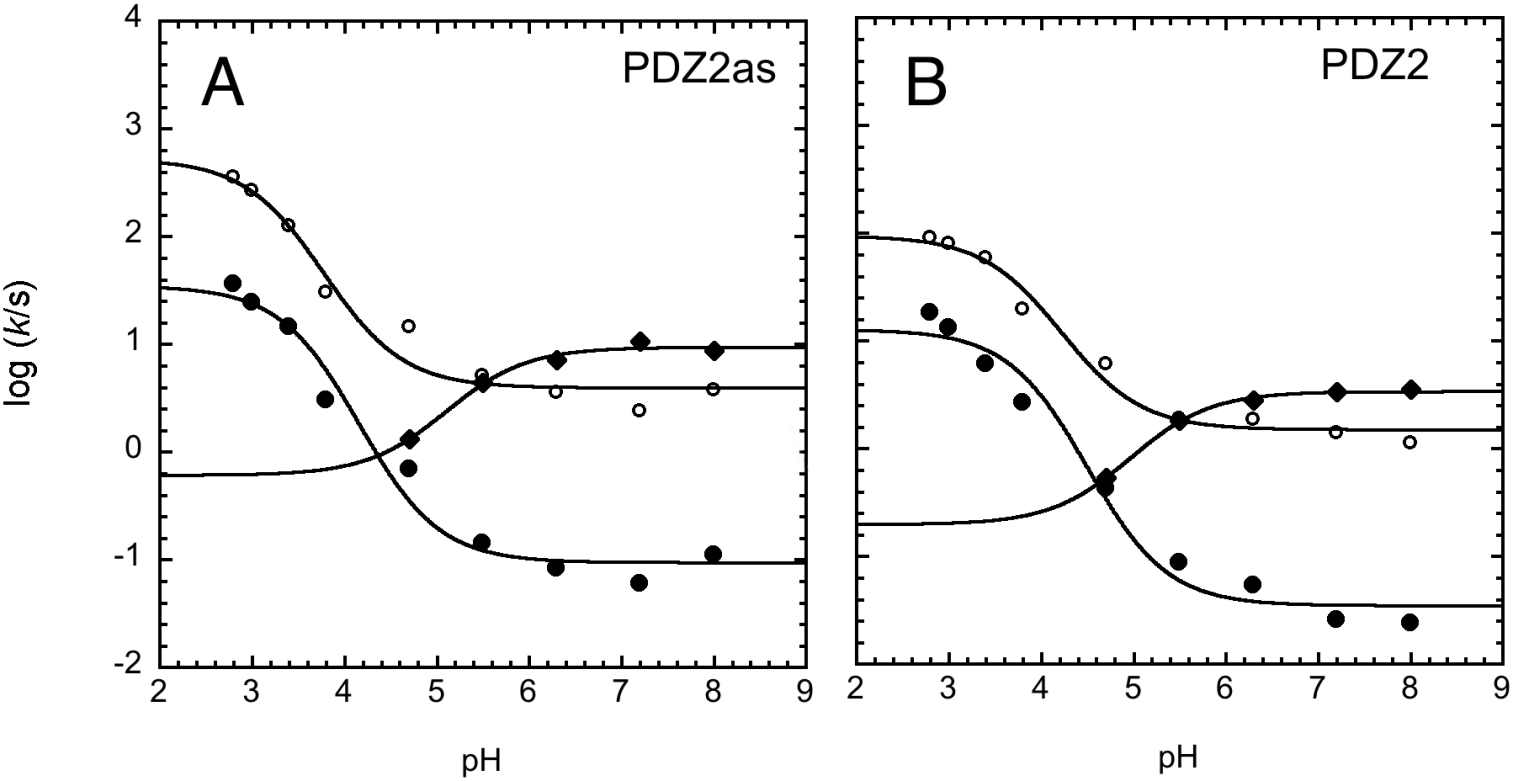
Dependence of the folding and unfolding rate constants of pWT43 PDZ2as and of pWT43 PDZ2 as a function of pH. The rate constants were calculated from a global analysis of the data in Figure 4 (pWT43 PDZ2as, panel A) and from the data reported in Ref. 34 (pWT43 PDZ2, panel B). The rate constant refer to the folding rate constant *k*_F_ (diamonds), and the two unfolding rate constant *k*_U1_ (filled circles) and *k*_U2_ (open circles), as detailed in the Materials and methods section.

By following a three-state model involving a high energy intermediate^39^, data in Figure 4 allow to calculate the folding rate constant k_F_ and two different unfolding rate constants *k*_U1_ and *k*_U2_ associated with the difference in free energy between the native states and two transition states, a more denatured like TS1 and a more native like TS2. The calculated values are listed in Table 1, together with a comparison between the thermodynamic stabilities for PDZ2as calculated from equilibrium transitions and kinetic experiments. Furthermore, it is possible to calculate the relative position of these experimentally accessible activation barriers along the reaction coordinate (namely the so-called Tanford β-value), resulting in a β-value of 0.55 ± 0.02 for the unfolded like transition state TS1 and 0.90 ± 0.05 for the native-like activation barrier TS2. These values are extremely similar to those obtained for pWT43 PDZ2, which returned values of 0.57 ± 0.03 for TS1 and 0.89 ± 0.03 for TS2^36^, indicating that whilst accelerating both folding and unfolding, the 5 amino acids insertion does not contribute to relevant distortion of the folding transition state structures. Furthermore, the dependences on pH of the microscopic rate constants is very similar for the two proteins (Figure 5), suggesting that the non-native interactions stabilizing the transition states in pWT43 PDZ2as versus pWT43 PDZ2 are not affected by changes in pH.

**Figure 6:**
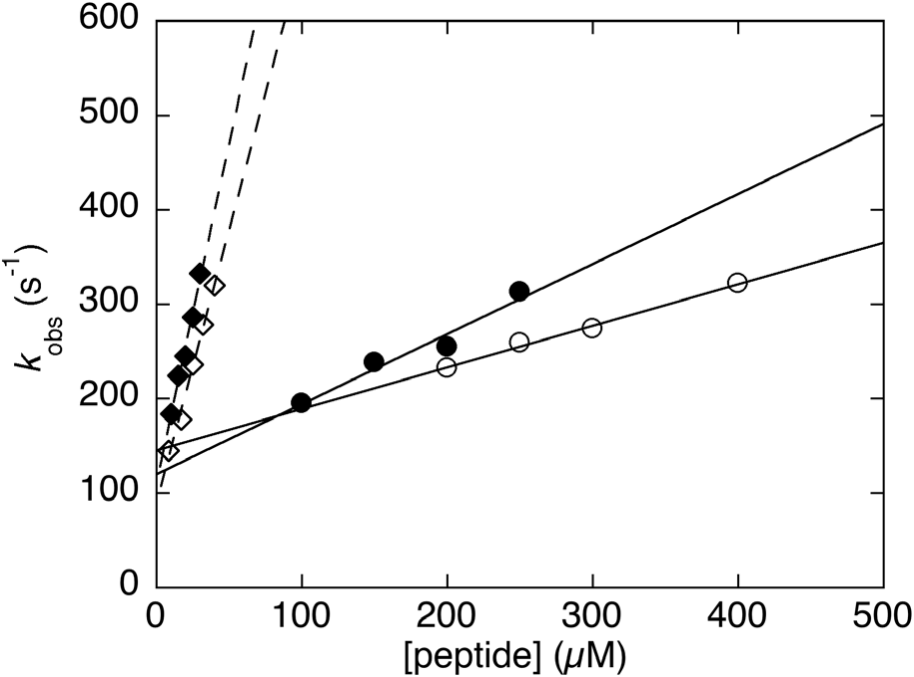
Binding properties of pWT43 PDZ2as and of pWT43 PDZ2. Pseudo first order kinetics of the binding between pWT43 PDZ2as (circles) and pWT43 PDZ2 (diamonds) carried out at pH 7.2, 25°C. Data refer to both the APC (open symbols) and RIL (filled symbols) peptide. It is evident that the alternative splicing affects binding by primarily lowering the association rate constant *k*_on_ (the slope of the observed dependences), with negligible effects on *k*_off_ (the intercept at concentration zero of peptide).

Previous studies have highlighted that the alternative splicing affects remarkably the binding function of the PDZ moiety, by nearly abrogating binding^27^,^28^. To address the binding mechanism of pWT43 PDZ2as and its physiological partners, we performed kinetic experiments at 25°C. We carried out kinetic binding experiments by subjecting to temperature jump kinetics a constant concentration of pWT43 PDZ2as (2 μM) in the presence of excess concentrations of dansylated peptides mimicking the PDZ binding motif of APC and RIL; the latter concentrations typically varying from 100 to 400 μM. In particular, individual solutions containing a constant concentration of PDZ2as 2 μM and different concentrations of APC and RIL peptide were prepared and, subsequently, each of these solutions was subjected to a rapid temperature-jump to explore sub-ms relaxation binding kinetics. Under all the explored conditions, the observed time course was satisfactorily fitted by a single-exponential function, without evidence for accumulation of detectable intermediates. Analysis of the pseudo-first order dependence of observed rate constants (Figure 6) yields an association bimolecular rate constant k_on_ of (0.44 ± 0.1) 10^6^ s^−1^ M^−1^ and (0.74 ± 0.1) 10^6^ s^−1^ M^−1^ and a dissociation rate constant k_off_ of 145 ± 10 s^−1^ and 120 ± 15 s^−1^, respectively for APC and RIL peptides. The overall K_D_ obtained from kinetic experiments are 352 ± 20 and 162 ± 10 μM, respectively for APC and RIL. As previously suggested by other groups^20^,^27^,^28^, these high values of K_D_ suggest the alternative splicing of PDZ2 to represent a mechanism to abrogate binding to the physiological partners in the cell. A comparison between the observed kinetics on the light of previously reported structural and kinetic data of pWT43 PDZ2 is briefly discussed below.

## Discussion

PDZ domains represent the most abundant mediators of protein-protein interactions in the human cell^1^,^2^,^3^. Because of the complexity of their role, their mechanisms of regulation may be rather complicated and span different scenarios. Among these, alternative splicing represents one of the most interesting features and, despite it has been observed on 8 different PDZ domains^2^,^40^, it has not been studied extensively to date, thus demanding additional characterization. Here we have compared the folding and binding properties of the PDZ2 with its alternative spliced counterpart, containing an insertion of 5 amino acids, and we will discuss below the key similarities and differences.

In an effort to provide a comprehensive description of the folding and stability of PDZ2as, we performed equilibrium and kinetic experiments under different experimental conditions. Interestingly, a comparison of the folding and unfolding rate constants of pWT43 PDZ2as versus pWT43 PDZ2 shows that, whilst the general features of the chevron plots are similar, the insertion of 5 amino acids in the β2–β3 loop induces the protein to fold and unfold about three times faster than pWT43 PDZ2*.* This observation suggests that the transition state of PDZ2as forms some stabilizing interactions that are not formed in its native state. In fact, while the transition state is stabilized, the rate constant for unfolding from the native state to the transition state is faster than in PDZ2. In protein folding studies, this type of behavior is classically interpreted as a signature for transient weak non-native interaction in the transition state, being formally similar to a negative phi value^41^,^42^,^43^,^44^. An alternative explanation would imply the insertion of five residues to contribute to more favorable contacts, thus replacing a poor interaction network in a transition state and, therefore, to stabilize its structure without changing its compaction. Remarkably, the robustness of the pH dependence of the calculated microscopic rate constants reported in Figure 4 demonstrates that none of the groups that are protonated between pH 2 and 8 is affected by the alternative splicing and, therefore, engaged in these non-native interactions.

It is of interest to compare the thermodynamic stabilities of PDZ2as versus PDZ2. In fact, whilst previous NMR characterization suggested the alternative splicing to affect the stability of the native state, our analysis of the difference in free energy between the native and denatured states of the two proteins suggests an apparent negligible change in stability. However, a comparison of the overall structural features of the denatured states of PDZ2as and PDZ2 may reconcile these apparently contrasting observation. In fact, the dependence of the calculated *m*-values, reported in Figure 3, clearly reveals a difference between the two proteins. This observation suggests that the apparent lack of changes in stability from urea induced denaturation experiments may arise from a complex effect, which implies the insertion of 5 amino acids in the β2–β3 loop to affect the free energies of both the native and denatured states.

Previous studies have demonstrated that the most remarkable effect induced by the alternative splicing lies in affecting the binding capability of the PDZ domain for both the APC and RIL peptides^20^,^27^,^28^. Furthermore, NMR characterization of the domain suggested this drop in affinity to arise at least in part from a closed binding pocket of the domain^28^, the structure of free PDZ2as resembling the structure of the “closed” PDZ2, observed in the complex with its ligand (Figure 1)^10^, an effect which is reminiscent of what previously observed on the *Drosophila melanogaster* acylphosphatase^45^. We have previously characterised extensively the binding between PDZ2 and both RIL and APC ligands and suggested the overall mechanism to occur via an induced fit type scenario, whereby the protein recognizes the peptide in an open conformation and then subsequently locks in a closed state^5^,^15^,^24^,^25^,^46^,^47^,^48^. This mechanism was drawn by employing a synergy between ultra fast mixing kinetics, site directed mutagenesis and NMR spectroscopy^10^. From this perspective, it is of interest to analyze the effect of alternative splicing on the observed reaction kinetics. In particular, the observed decrease of the microscopic association rate constant, with negligible effect on the dissociation rate constant, appears to conform to the earlier observation indicating PDZ2as to populate a closed conformation. In fact, under such conditions, in line with an induced fit scenario, the protein would need to open before binding to occur and, accordingly, the association rate constant would decrease by a factor equal to (1 + K_eq_), where *K*_eq_ is the equilibrium constant between the closed and open form in unbound PDZ2as. Future work based on site-directed mutagenesis will further help in dissecting the molecular details of the effect of the alternative splicing in the thermodynamics of folding and binding of PDZ2.

## Methods

### Site-Directed Mutagenesis and Protein Expression and Purification

pWT43 PDZ2as, the alternatively spliced form of pWT43 PDZ2, was obtained by using the Quick-change mutagenesis Kit (Stratagene), according to the manufacturer's instructions. The mutations, an insertion of 5 residues (VLFDK) in position 30 at the beginning of the β2–β3 loop, were confirmed by DNA sequencing.

pWT43 PDZ2 and pWT43 PDZ2as were expressed and purified as described previously^23^,^25^.

Dansylated peptides corresponding to the C-terminus of APC and RIL, sequences D-SYLVTSV and D-EQVSAV, respectively, were purchased from JPT Peptide Technologies (Berlin, Germany); peptides were purified using high-performance liquid chromatography-purified peptide with purity higher than 95%.

### Equilibrium unfolding

Equilibrium denaturations were carried out on Fluoromax single photon counting spectrofluorometer (Jobin-Yvon, Edison, NJ). Tryptophan fluorescence emission spectra were recorded in a cuvette (1-cm light path) between 300 and 400 nm. The excitation wavelength was 280 nm. Protein concentrations were typically 3 μM. The buffer used was 50 mM sodium phosphate pH 7.2 at different temperatures ranging from 5°C to 35°C.

### Stopped-flow measurements

Single mixing kinetic folding experiments were carried out on an SX-18 stopped-flow instrument (Applied Photophysics, Leatherhead, UK); the excitation wavelength was 280 nm and the fluorescence emission was measured using a 320 nm cut-off glass filter. The experiments were performed at 25°C and the buffer used were: 50 mM Tris/HCl pH 8.0, 50 mM sodium phosphate from pH 7 to 6.3, 50 mM sodium acetate from pH 5.5 to 3.8, 50 mM sodium formate from 3.4 to 2.8, 50 mM sodium phosphate pH 2.1. All reagents were of analytical grade.

### Temperature-jump fluorescence spectroscopy

Binding experiments were performed mixing a constant concentration of PDZ2as 2 μM with increasing concentrations of the APC or RIL peptide ranging from 100 to 400 μM in 50 mM sodium phosphate pH 7.2, in the presence of 150 mM KCl. The experiments were carried out using a Hi-Tech PTJ-64 capacitor-discharge T-jump apparatus (Hi-Tech, Salisbury, U.K.). Temperature was rapidly changed from 16°C to 25°C with a jump of 9°C. 20 individual traces were averaged at given peptide concentration. The fluorescence change of N-acetyltryptophanamide (NATA) was used in control measurements. Protein concentration was typically 20 μM. The excitation wavelength was 296 nm and the fluorescence emission was measured using a band pass 315 +/− 25 nm glass filter.

### Data analysis

Equilibrium experiments. Assuming a standard two-state model, the urea induced denaturation transitions were fitted to the equation:
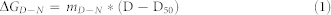
where Δ*G*_D-N_ is the free energy of folding in water and at a concentration D of denaturant, m_D-N_ is the slope of the transition (proportional to the increase in solvent-accessible surface area in going from the native to the denatured state) and D_50_ is the midpoint of the denaturation transition. An equation that takes into account the pre- and post-transition baselines has been used to fit the observed unfolding^39^.

In analogy with previous experiments of PDZ domains^25^,^35^,^36^,^37^, the folding chevron plots were fitted to a model implying a high-energy intermediate, intervening between two consecutive transition states, namely a more denatured like TS1 and a more native-like TS2. Under such condition the observed rate constant would be equal to:
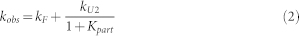
where *k*_F_ is the folding rate constant, *k*_U2_ is the unfolding rate constant from the native to the second transition state TS2, and *K*_part_ is the partition factor between the two transition states. By following this model, a second unfolding rate constant *k*_U2_, which represent a combined rate constant approximating the unfolding from the native to the denatured like TS1, may be calculated by multiplying *k*_U2_ for the partition factor *K*_part_.

## Author Contributions

S.G. planned the research project; E.D.S., A.T., D.B. and A.M. conducted the experiments; all the authors analyzed data; S.G. wrote the main manuscript text and prepared figures, and all authors reviewed the manuscript.

## Figures and Tables

**Table 1:** Kinetic folding parameters of PDZ2 from PTP-BL as a function of pH calculated in water

pH	*k*_F_ (sec^−1^)	*k*_U1_ (sec^−1^)	*k*_U2_ (sec^−1^)	*K*_part_	Δ*G*_D-N_ a (kcal M^−1^)	Δ*G*_D-N_ b (kcal M^−1^)
8.0	8.8 ± 0.1	0.11 ± 0.001	3.75 ± 0.9	0.029 ± 0.005	2.58 ± 0.05	2.2 ± 0.3
7.2	10.7 ± 0.2	0.060 ± 0.002	2.40 ± 0.04	0.025 ± 0.05	3.05 ± 0.07	2.4 ± 0.2
6.3	7.2 ± 0.4	0.082 ± 0.008	3.57 ± 0.008	0.023 ± 0.004	2.64 ± 0.09	2.1 ± 0.2
5.5	4.43 ± 0.2	0.14 ± 0.01	5.05 ± 0.05	0.028 ± 0.01	2.04 ± 0.08	1.8 ± 0.3
4.7	1.3 ± 0.2	0.70 ± 0.02	14.5 ± 0.01	0.048 ± 0.01	0.7 ± 0.1	1.0 ± 0.3
3.8	-c	3.04 ± 0.08	30 ± 0.3	0.10 ± 0.01	-d	-d
3.4	-c	14.56 ± 0.09	126 ± 2	0.11 ± 0.02	-d	-d
3.0	-c	24.3 ± 0.5	266 ± 3	0.09 ± 0.01	-d	-d
2.8	-c	36 ± 1	352 ± 40	0.10 ± 0.01	-d	-d

^a^Calculated from kinetic experiments;

^b^Calculated from equilibrium experiments.

Experimental data were fitted to a three-state model as formalized in Eq. 2. Standard errors are reported. The Tanford β-value obtained from chevron analysis for the two transition states were β = 0.55 ± 0.02 for TS1 and β = 0.90 ± 0.05 for TS2. These values are extremely similar to those obtained for pWT43 PDZ2, which returned values of 0.57 ± 0.03 for TS1 and 0.89 ± 0.03 for TS2^36^.

^c^The protein was too unstable to determine folding parameters.

^d^The protein was too unstable to determine reliable stability parameters.
